# Preconception care: caffeine, smoking, alcohol, drugs and other environmental chemical/radiation exposure

**DOI:** 10.1186/1742-4755-11-S3-S6

**Published:** 2014-09-26

**Authors:** Zohra S Lassi, Ayesha M Imam, Sohni V Dean, Zulfiqar A Bhutta

**Affiliations:** 1 Division of Women and Child Health, Aga Khan University Karachi, Pakistan

**Keywords:** preconception, overweight, folic acid, nutrition

## Abstract

**Introduction:**

As providing health education, optimizing nutrition, and managing risk factors can be effective for ensuring a healthy outcome for women and her yet un-conceived baby, external influences play a significant role as well. Alcohol, smoking, caffeine use and other similar lifestyle factors, have now become an integral part of the daily life of most men and women, who use/misuse one or more of these harmful substances regularly despite knowledge of their detrimental effects. The adverse health outcomes of these voluntary and involuntary exposures are of even greater concern in women of child bearing age where the exposure has the potential of inflicting harm to two generations. This paper is examining the available literature for the possible effects of caffeine consumption, smoking, alcohol or exposure to chemicals may have on the maternal, newborn and child health (MNCH).

**Methods:**

A systematic review and meta-analysis of the evidence was conducted to ascertain the possible impact of preconception usage of caffeine, tobacco, alcohol and other illicit drugs; and exposure to environmental chemicals and radiant on MNCH outcomes. A comprehensive strategy was used to search electronic reference libraries, and both observational and clinical controlled trials were included. Cross-referencing and a separate search strategy for each preconception risk and intervention ensured wider study capture.

**Results:**

Heavy maternal preconception caffeine intake of >300mg/d significantly increase the risk of a subsequent fetal loss by 31% (95% CI: 8-58%). On the other hand, preconception alcohol consumption leads to non-significant 30% increase in spontaneous abortion (RR 1.30; 95% CI: 0.85-1.97). Preconception counselling can lead to a significant decrease in the consumption of alcohol during the first trimester (OR 1.79; 95% CI: 1.08-2.97). Periconception smoking, on the other hand, was found to be associated with an almost 3 times increased risk of congenital heart defects (OR 2.80; 95% CI 1.76-4.47). While the review found limited evidence of preconception environmental exposure on maternal, newborn and child health outcomes, occupational exposure in female radiation workers before conception showed an increased impact in risk of early miscarriages.

**Conclusion:**

Identification of substance abuse and environmental history during preconception period provides an opportunity to assist women in reducing major health risks and identify key determinants of healthy pregnancy. Studies have shown that the aversion and prevention of exposure feasibility can play an important role in improving the health of women and their families, however, the results should be interpreted with great caution as there were few studies in each section. Therefore, there is a need for more rigorous studies to test the hypotheses.

## Introduction

While factors like maternal nutritional status, disease control and reproductive planning are critical in ensuring a healthy outcome for women and their future offspring, exposure to drugs and chemicals play a significant role as well. Alcohol, smoking, caffeine use and other similar lifestyle factors, have now become an integral part of the daily life of most men and women, who use/misuse one or more of these harmful substances regularly despite knowledge of their detrimental effects. Exposure to reproductive and developmental toxicants in the environment (at home and at the workplace) is of concern. The adverse health outcomes of these exposures are of even greater concern in women of child bearing age where the exposure has the potential to inflict harm to her baby in utero as well.

Alcohol is a known teratogen and prenatal alcohol use is an important preventable cause of maternal morbidity and neonatal birth defects and developmental disabilities. A recent National Survey on Drug Use and Health [1] quoted rates of current alcohol use to be 57% in adult females and rates of binge drinking (≥5 drinks on the same occasion) to be approximately 25% in the United States. Smoking is another factor that leads to known maternal and fetal complications during pregnancy and almost one third of women of child-bearing age smoke [1]. Exposure to caffeine during pregnancy has been associated with an increased risk of spontaneous abortion and low birth weight [2]. Often, these habits begin during adolescence, only to be consolidated as young men and women become adults. Many young women continue to be exposed to these harmful substances during the first trimester without realizing they are pregnant, exposing the fetus to the toxic effects of substance use during organogenesis, resulting in long-lasting effects on the child’s physical and mental development. Alcohol, tobacco and illicit drug use are often so deeply engrained that many continue use even during pregnancy as shown by the National Survey of Drug Use and Health (NSDUH) survey. The survey reported that almost 11% of the pregnant women stated current alcohol use and 5% of pregnant women use some form of illicit drugs [1]. More than 80% of pregnant women also attested to consuming some form of caffeine in another study [3]. Alongside these, there are environmental and occupational hazards with teratogenic potential and a woman’s professional and residential activities may pose risks for her before pregnancy. These are not limited to work-related exposures to radiation, but also encompass contact with substances like lead in paints, mercury from consumption of seafood, or pesticides in the soil.

These risks and the interventions to prevent and avoid them are an important component of preconception care. In this review, preconception care is defined as “any intervention provided to women and couples of childbearing age, regardless of pregnancy status or desire, *before* pregnancy, to improve health outcomes for women, newborns and children”. Periconceptional care, on the other hand, is any intervention provided to women of childbearing age preceding conception but that continues into the first trimester, to improve health outcomes for women, newborns and children [4].

This systematic review was conducted to synthesize the evidence linking each of these potentially avoidable risk factors during preconception period and their impact on maternal, newborn and child health (MNCH) outcomes. The review also identified and assessed the impact of interventions for avoiding the usage of caffeine, tobacco, alcohol and other illicit drugs; and exposure to environmental or workplace chemicals and radiation that have shown promising results on MNCH.

## Methods

This paper systematically reviewed all the literature published up to 2011 to identify studies describing the effectiveness of preconception risks and intervention to prevent and avoid substance abuse and environmental and workplace exposure to chemicals and radiations for improved maternal, newborn and child health (MNCH) outcomes. Electronic databases such as PubMed, Cochrane Libraries, Embase, and WHO Regional Databases were searched to identify the experimental and observational studies on the subject. Papers were also identified by hand searching references from included studies. No language or date restrictions were applied in the search. The findings were presented at international meeting [5,6]and shared with professionals in the relevant fields of maternal and child health, following which results were updated based on current searches (through end of 2012) and expert opinion. Studies were included if they reported the effectiveness of interventions for and risks to substance abuse and environmental exposure on MNCH outcomes. The methodology is described in detailed elsewhere [4].

Two authors assessed the eligibility of studies and extracted data and judged the quality on standardized sheets. The quality of experimental studies were assessed using Cochrane criteria [7], whereas STROBE guidelines were used to assess the quality of observational studies [8]. We conducted meta-analyses for individual studies and pooled statistics was reported as the odds ratio (OR) and relative risk (RR) between the experimental and control groups with 95% confidence intervals (CI). Mantel–Haenszel pooled RR and corresponding 95% CI were reported or the Der Simonian–Laird pooled RR and corresponding 95% CI where there was an unexplained heterogeneity. Since most of the studies were observational in design, therefore, we used generalized inverse variance (GIV) method to pool data which is a better way to run a comparative analysis [9]. All analyses were conducted using the software Review Manager 5.1 [10]. Heterogeneity was quantified by Chi^2^ and I^2^, in situations of high heterogeneity, causes were explored through sub-group analysis and random effect models were used.

## Results

**Figure 1:**
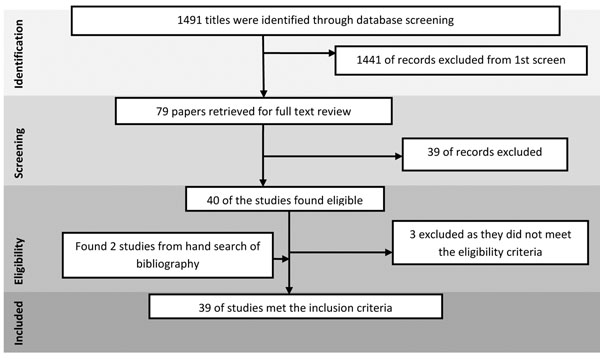
Search flow diagram

The search identified 1491 papers. After the initial title and abstract screening, 40 full texts were reviewed to assess which papers met the inclusion criteria and had the outcomes of our interest. Thirty nine studies were finally selected for abstraction and analysis (Figure 1). On quality assessment, observational studies often had inadequate description of the quantitative exposure, and instead categorized exposure as high versus low with variable cutoffs used in each study. Studies had limited quality with respect to statistical analysis and adjustment for confounders. Overall studies were of moderate quality. The quality of intervention studies was compromised with respect to selection, performance and reporting bias. Information related to each included study can be found on the following link:

https://globalmotherchildresearch.tghn.org/site_media/media/articles/Preconception_Report.pdf

### Periconceptional caffeine intake

With regards to preconception caffeine intake, some studies have concluded that a high daily intake of caffeine prior to pregnancy leads to an increased risk of spontaneous abortion [2,11,12] while others reported no association [13-15].

**Figure 2:**
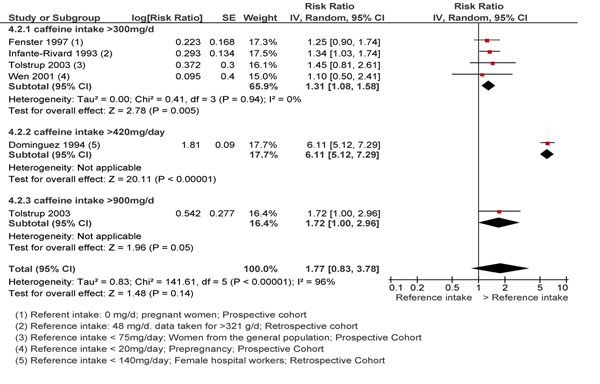
Periconception caffeine intake and risk of spontaneous abortions: evidence from observational studies

A total of six observational studies (3 prospective and 2 retrospective) [2,11,12,16-18] were identified on the effect of preconception caffeine intake on fetal loss. Of these, 5 were included in meta-analysis as one study [11] failed to provide data on the quantity of caffeine ingested. The review categorized the level of caffeine intake into >300mg/day, >420mg/day and >900mg/day and compared with an intake <150mg/day. Caffeine intake was found to be significantly associated with fetal loss: >300mg/day (RR 1.31; 95% CI: 1.08-1.58), intake >420mg/day (RR 6.11; 95% CI: 5.12-7.29) and >900mg/day (RR 1.72; 95% CI: 1.00-2.96). Although the forest plot cannot be interpreted for a dose-response relationship due to limitations with regards to a comparison group (mentioned in the forest plot), each individual study had reported a non-significant increase in risk of spontaneous abortion with daily increased caffeine consumption (Figure 2).

### Alcohol intake

Prenatal alcohol exposure is associated with spontaneous abortion, prenatal and postnatal growth restriction and birth defects [19-21]. It is one of the leading causes of neurodevelopmental deficits in children in the United States, as a result of fetal alcohol spectrum disorder (FASD) [22-26].

Population-based behavior modification interventions (reduction of alcohol use and improved contraception) in women of child-bearing age show promise [27-29]. Content of preconception care for reducing alcohol intake includes screening of all women of reproductive age, intervention for those who are consuming it excessively (>7 drinks/week or > 3 drinks on 1 occasion), counseling about the adverse effects on pregnancy outcomes, promotion of abstinence programs for those with alcohol dependency and encouragement of consistent contraceptive use for those not in control of their drinking habits.

**Figure 3:**
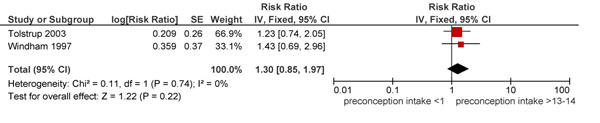
Preconception alcohol intake and risk of Spontaneous abortions: evidence from observational studies

The review identified eight [2,19,30-36] risk aversion and two intervention studies [29,37] dealing with both pre- and periconception drinking as well as interventions during these same periods to alter drinking behavior among women. The analysis showed a non-significant 30% increase in the risk of occurrence of spontaneous abortions with preconception alcohol consumption (Figure 3).

Another study [30] displayed a non-significant increased odds of neural tube defects (NTDs) with preconception alcohol intake (OR 1.24; 95% CI: 0.92-1.68), while preconception binge drinking was associated with 20% greater risk of NTDs when compared with alcohol intake of 1 drink/day. Periconception alcohol consumption was also found to be associated with esophageal atresia with or without tracheo-esophageal fistula (RR 1.26; 95% CI: 1.03-1.56). While Carmichael et al. [33] did not find any association of periconceptional alcohol exposure with congenital heart defects (OR 0.96; 95% CI: 0.91-1.01), Mullany et al. [38] displayed a non-significant increased risk of conotruncal heart defects with alcohol intake once weekly (RR 1.6; 95% CI: 0.8-3.0) compared to none..

The review also found an association of preconception substance abuse with depression [36]. Analysis showed higher rate of depression among alcohol users however the rates of depression increased with combined use of alcohol and other drugs compared to when alcohol was taken alone (RR 1.94; 95% CI: 1.38-2.73).

**Figure 4:**
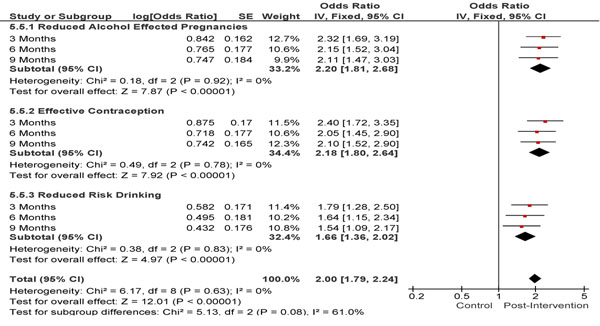
Post intervention reduction in risk drinking and improvement in effective contraception use, thereby leading to a reduction in Alcohol pregnancy: evidence from controlled trial

Preconception counselling [37] led to a significant decrease in the consumption of alcohol during the first trimester (OR 1.79; 95% CI: 1.08-2.97) however the decrease in binge drinking episodes (OR 1.51; 95% CI: 0.63-3.62) was not significant. There was no association of preconception counselling and continuing to drink through the rest of pregnancy (OR 0.95; 95% CI: 0.59-1.53). Another paper [29] found an impact on reducing the rates of alcohol exposed pregnancy (AEP) amongst those who consumed more than 8 drinks per week through motivational interviewing. On three months follow up the odds of being at reduced risk of AEP was 2.31 (95% CI 1.69-3.20); at 6 months the odds was 2.15 (CI1.52-3.06); and at 9 months the odds was 2.11 (CI1.47-3.03). This intervention also led to a significant increase in the use of effective contraception (OR 2.18; 95% CI: 1.80-2.64) thereby significantly reducing the risk of an AEP (OR 2.20; 95% CI: 1.81-2.68). This finding was in accordance to the results of another trial [28] of a behavioural intervention where 74% of women were no longer at risk for AEP at 1 month post-intervention (Figure 4).

### Pre and Peri conception smoking

Fetal effects of exposure to maternal smoking include intrauterine growth restriction (IUGR), prematurity, LBW, congenital malformations [39-44] and sudden infant death syndrome [45]. In the past two decades many clinical trials have demonstrated the effectiveness of smoking cessation interventions in early pregnancy[46].

The content of preconception care for women who smoke includes: screening of all women of child-bearing age using evidence-based guidelines, treatment of dependence before planning a pregnancy, informing women about the adverse pregnancy outcomes associated with tobacco consumption, discussion of possible interventions to assist in quitting (including the use of medications) and referring to intensive counseling services where necessary.

The review identified seven risk aversion studies [30,32,34,35,47-50] and one intervention study [37] on the effect of preconception smoking cessation. A study by Haas et al. [47] showed that preconception smoking was significantly associated with preterm births (OR 2.2; 95% CI: 1.29-3.75). On the other hand, no impacts were seen on orofacial defects (OR 1.17; 95% CI: 0.89-1.52) [32,34,48] and NTDs whether the consumption was greater or less than a pack per day (OR 0.80; 95% CI: 0.59-1.08) [30].

**Figure 5:**
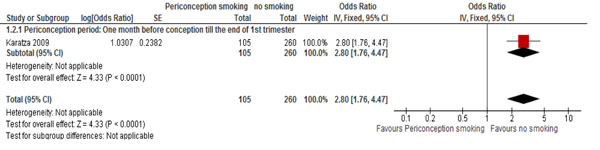
Periconception maternal smoking and risk of congenital heart defect: evidence from observational studies

Periconception smoking was significantly associated with 3 folds increased risk of congenital heart defects (OR 2.80; 95% CI 1.76-4.47) (Figure 5) [49], while no association was found between exposure and esophageal atresia with or without tracheoesophageal fistula (RR 0.95; 95% CI: 0.76-1.19) [31] and congenital diaphragmatic hernia (OR 1.10; 95% CI: 0.91-1.33) [35].

On comparing light smoking (<14 cigarettes/day) with heavy (>25 cigarettes/day) in the periconception period (1 month before pregnancy till the end of the 1^st^ trimester), light smoking was associated with non-significant 17% lower risk of heart defects than heavy smoking (RR 0.83; 95% CI: 0.65-1.07) [32]. On assessing the effect of preconception parental smoking on the risk of childhood leukemia, it was seen that paternal smoking was significantly associated with a greater risk (OR 0.99; 95% CI: 0.66-1.47), whereas, maternal smoking was not associated with risk of developing leukemia in the childhood period (OR 0.99; 95% CI: 0.66-1.47) [50].

The review identified one intervention study that compared the effectiveness of preconception counseling with standard care and found 3 times greater likelihood of women quitting smoking in the post-intervention group, although the association was not statistically significant (2.94; 95% CI: 0.70-12.36) [37].

### Illicit drugs consumption

Maternal substance abuse is associated with pregnancy complications, LBW, an increased risk of infant mortality, neonatal abstinence syndrome, ineffective parenting techniques, child abuse and neglect, and possible increased likelihood of human immunodeficiency virus (HIV) transmission. Substance abuse also is often associated with other social and health problems that affect both the mother and infant, including domestic violence, poverty, homelessness, sexual abuse, psychiatric disorders, and poor health care [51].

The review aimed to assess the effects of preconception abuse of illicit drugs and possible interventions to reduce the level of abuse amongst women of reproductive age before conception Preconception care includes identification of women who are consuming illicit drugs, counseling on the risks associated with preconception use of various drugs, informing about programs that support abstinence and treatment and promoting the importance of contraception until cessation of use is achieved.

**Figure 6:**
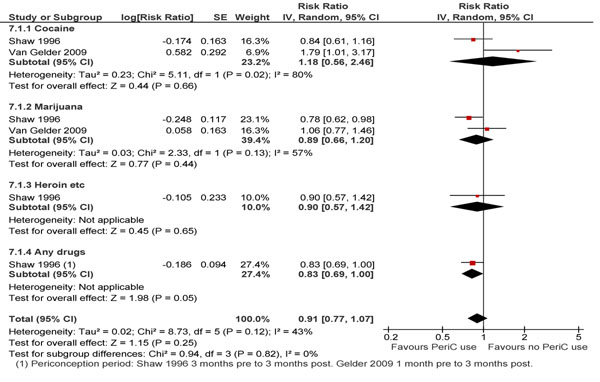
Maternal periconception consumption of illicit drugs and risk of NTDs: evidence from observational studies

The review found a limited number of risk aversion [30,36,52,53] and intervention studies [54,55] concerned with the MNCH effects of periconception substance abuse. Paternal periconception use of illicit drugs overall did not have an association with the risk of NTDs (RR 1.07; 95% CI: 0.87-1.31) [30], however a significant association was found with heroin use alone (RR 1.63; 95% CI: 1.23-2.16). Maternal use of recreational drugs during the periconception period had no impact on occurrence of NTDs (RR 0.91; 95% CI: 0.77-1.07) [30,52] (Figure 6). Comparing parental use (combined or individual) versus no use did not yield any significant association with NTD risk [30].

A strong association was found between recreational drug use in the month in which conception occurred and incidence of gastroschisis, although this evidence came from a single case-control study (OR 1.76; 95% CI: 0.99-3.13) [36]. A significant association was also found between substance abuse before pregnancy and maternal depression (OR 9.60; 95% CI: 1.80-51.20) [53].

Amongst the two interventional studies, one studied the health seeking behavior of substance-abused women using the ‘Steps of Change’ model [54]. The other compared the effect of behavioral couple’s therapy (BCT) with individual-based therapy (IBT) amongst substance-abusing men and assessed its impact on incidence of partner violence [55]. It reported that post-intervention, male-to-female aggression in the BCT group was lower than in the IBT group (17% vs. 43%). Also husbands in the BCT group reported fewer days of drug use, longer episodes of abstinence, and less drug-related hospitalizations or arrests.

## Ameliorating environmental exposures

### Radiation exposure

Ionizing radiation is known to have harmful effects on the reproductive systems of both males and females [56]. It is one of the few known factors to increase the likelihood of childhood cancers. The danger to the embryo and fetus of intrauterine ionizing radiation exposure is well documented [57,58]. While some studies have found a significant association between preconception paternal exposure to ionizing radiation and non-Hodgkin’s lymphoma in their children [59-61] others found a weak, non-significant association [62] or no association at all [63-67]. Studies have also shown a significant elevated risk of childhood cancers in children born to exposed female radiation workers [60,61].

Ionizing radiation exposure is composed of both, occupation-related exposure as well as non-occupational exposure. The content of preconception care with respect to environmental exposures consists of taking a detailed history of the couple to identify possible sources of radiation exposure, informing women of child-bearing age about the possible deleterious effects (miscarriage, stillbirths, childhood cancer) of such exposure, on their health as well as that of the fetus, especially during the time where a woman does not yet know she is pregnant..

Both occupational [62,68,69] and non-occupational [33,34,70] radiation exposure were analyzed separately. One reported the effect of preconception occupational radiation exposures in women on fetal death [68]. This study showed an increased risk of early miscarriage in mothers who were exposed to radiation in the workplace (RR 1.32; 95% CI: 1.04-1.66). This association was stronger in women who were monitored within 6 months of conception (RR 1.50; 95% CI: 1.20-1.87) . There was also a non-significant association with stillbirths a (RR 2.30; 95% CI: 0.80-6.62) and second trimester miscarriage (RR 0.50; 95% CI: 0.20-1.26) [68].

**Figure 7:**
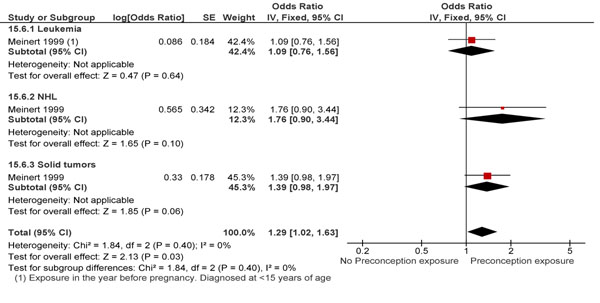
Preconception maternal exposure to radiation and childhood cancer: evidence from observational studies

**Figure 8:**
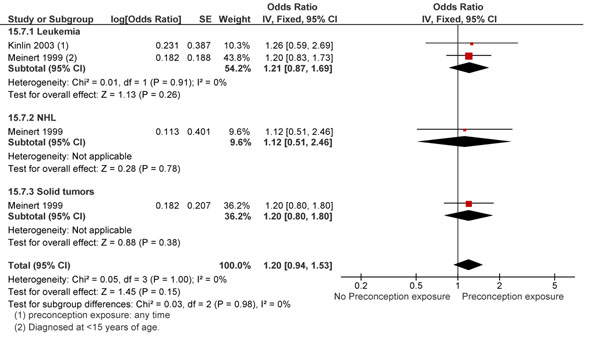
Preconception paternal exposure to radiation and childhood cancer: evidence from observational studies

Both paternal and maternal exposure to preconception ionizing radiation at work led to an overall greater risk of childhood cancers (RR 1.29; 95% CI: 1.02-1.63; and RR 1.19; 95% CI: 0.92-1.54 respectively) (Figure 7 and Figure 8), however this association was only significant for maternal exposure. This finding is not in conjunction with the results of Bunch et al. [71] on the effect of preconception maternal radiation exposure on all childhood cancers (RR 1.90; 95% CI: 0.84-4.58). However, on pooling these studies, a significant association between exposure and outcomes was found (RR 1.33; 95% CI: 1.06-1.67). Studies looking at the effect of radiation dose on the incidence of childhood hematological malignancies had equivocal results [60,62,67,72-74]. However, many had samples too small to comment on the significance of their findings.

Non-occupational exposure to ionizing radiation via X-rays is associated with higher rates of adverse fetal and neonatal outcomes. Paternal exposure is associated with low mean birth weight (MD -73.00; 95% CI: -78.97, -67.03) [70] and reduced intrauterine growth (MD -53.00; 95% CI: -58.21, -47.79) [70]. There was no data available for possible effects of maternal preconception exposure on these outcomes. Parental X-ray exposure before conception also had an association with childhood cancers, especially between paternal abdominal exposure and leukemia in the offspring [62].

### Chemical exposure

An increasing body of scientific research provides disconcerting verification of the potential impact of environmental toxins that greatly affect human reproductive health and human development. It is believed that roughly 3% of fetal developmental defects are attributable to chemical exposures [75]. Toxic chemical exposures include organic solvents, metals, pesticides, polychlorinated biphenyl (PCB). Adverse outcomes associated with chemical exposures include LBW [76], spontaneous abortion, preterm birth, stillbirth/infant death, congenital anomalies [77], developmental delays [78], and childhood cancers [79].

This section reviewed literature for effects of exposure among either parent in the preconception period on the subsequent pregnancy and its outcomes. Early spontaneous abortions were defined as <12 weeks of gestation; >12 weeks but <20 weeks of gestation was classified as late spontaneous abortion. The content of preconception care with respect to environmental exposures consists of taking a detailed history of the couple to identify possible sources of toxic exposure, informing women of child-bearing age about the deleterious effects of such exposure, at home or at the workplace, on their health as well as that of the fetus, in case of an unknown pregnancy status.

The review found very limited evidence [80-89] regarding parental toxic exposure and effects on MNCH outcomes. Preconception exposure to pesticides was associated with 27% increase in spontaneous abortions (p <0.001); 31% increase in early spontaneous abortions (p=0.0007) and 22% increase in late spontaneous abortions (>12 weeks) (p=0.03) [80]. Paternal exposure to pesticides in the year before conception also showed an increase in the rates of hematological malignancies in their offspring [90-94].

Analysis of studies on parental exposure to chemicals like paints, solvents, industrial products etc. showed a 10% (p=0.02) increase in the risk of acute lymphoblastic leukemia (ALL) in subsequent offspring , similarly, the risk of ALL was 44% higher with maternal exposure (p<0.001) [81,82]. McKinney et al. [83] also found that exposure to dermal hydrocarbons and metal during the preconception period was linked to an increased risk ofleukemia and ALL. Vincetiet al. [84] reported an excess risk of cardiovascular defects (OR 2.59; 95% CI: 1.68-3.82) in a lead-polluted area.

Women who reported using wood to cook during the the periconception period had 3 folds increased risk of having infants with a NTD (95% CI:1.70-6.21) [85]. Another study reported that women who used wood, coal or tires in their home for cooking or heating had twice the odds of having a child with anencephaly compared with women not using this kind of fuel (OR 2.04; 95% CI: 1.29-3.23) [86].

Another area of unavoidable environmental exposure is that of traffic-related particulate air pollution. Perin et al.[87] showed that the risk of early pregnancy loss in women exposed to high levels of ambient particulate matter (>56.72 mg/m3) during the preconceptional period was almost 3-fold higher compared to women exposed to lower levels. The review also found evidence on the effect of bed-heating devices on the occurrence of certain birth defects (NTDs and orofacial defects), none of which were significant [88].

## Discussion

A growing number of women in their reproductive years continue to consume caffeine, alcohol, tobacco or illicit drugs. Moreover, increasing evidence from observational and epidemiological studies is supporting the linkages of harmful environmental exposures with human health and reproduction. While it is distressing that women (and men) continue to put themselves at risk, it is therefore important that measures should be taken to ameliorate these risk factors. This review found that heavy preconception caffeine consumption of >300mg/d significantly increased the risk of subsequent fetal loss by 31%. However, the association and mechanism by which caffeine intake affects MNCH outcomes is still not understood. Further research is also needed to assess the link between pre-gestational use of caffeine and spontaneous abortion. More specific research may help to elaborate on this in order to find a relatively safe cut-off. Preconception care, therefore, requires early identification of women who consume caffeine more than occasionally. Such women need to be informed of the potential risks of adverse pregnancy outcomes associated with caffeine intake (spontaneous abortion, low birth-weight). Cutting down to minimal or no caffeine intake should be the goal well in advance of a conception.

Although a positive pregnancy test may lead to a significant decrease in the alcohol use of many women, the intervening time lapse between that and conception are critical periods of fetal susceptibility to alcohol while the development of major organs is occurring. Alcohol consumption before or around the time of conception is linked to multiple adverse fetal outcomes including spontaneous abortion, gastrointestinal malformations and NTDs. None of these associations reach a level of statistical significance. Pre-pregnancy alcohol consumption is also correlated with maternal depression. Both preconception counselling as well as behavioural interventions have led to a significant improvement in drinking behaviour and thus pregnancies affected by alcohol. It is now required to use this important information to upgrade these interventions to have stronger, longer-lasting and more widespread effects.

Women, who are habitual smokers, put their health and that of their unborn child in jeopardy, as preconception and peri-conception smoking significantly increase the threat of preterm births by twice as much and also raises the risks of congenital defects in the fetus. In order to reduce the chances of the adverse effects delineated above, effective interventions for women of reproductive age who smoke should be implemented during preconception period and at a large scale. Although preconception counseling does improve practices in women pertaining to their smoking habits, behavioral interventions in synergy with general preconception counseling may play a more significant part. Hence it is important to motivate women of reproductive age to quit smoking before they conceive. Substantial research literature exists for interventions to quit smoking cessation among adults, women in general and pregnant women, however there is a dearth of clinical studies focusing specifically on non-pregnant women of childbearing age.

On the other hand, the current literature available to study the effect of preconception parental exposure to ionizing radiation on MNCH outcomes is limited, especially for fetal and early neonatal effects. The analyses showed some association of maternal exposure to radiation with early miscarriages and childhood cancers, and paternal exposure to X-ray with increased cases of childhood cancer.

In conclusion, there was a paucity of literature on the MNCH effects of exposure to various environmental agents in the preconception period. Although our data suggests a significant effect of preconception pesticide exposure on all spontaneous abortions and of preconception exposure to other chemicals (solvents, paints, industrial dust etc.) on the risk of ALL in their offspring, these results should be interpreted with great caution as only a small amount of relevant studies were pooled and analyzed. This clearly delineates the work for future research as countless environmental agents still need to be studied for their effects on MNCH outcomes. Only then will comprehensive interventions to reduce exposure be designed and evaluated for a final formation of a sound policy dealing with this topic. They should be advised to avoid eating shark, swordfish, king mackerel, and tile fish to reduce possible exposure to mercury. Women with a history of lead poisoning should be counseled on the risk on the unborn child.

The review found paucity of studies conducted in preconception period and reported MNCH outcomes. The number of experimental studies was too few and majority of the included studies were observational in nature. Although the association was studied well, there were small number of studies in each section and their quality was compromised. Therefore, the results from this review should be interpreted with great caution. The complete description of the exposure was also not reported in each study and the categorization of dosage of intake and severity of exposure varied across all the studies. Another limitation was the description of timing of exposure. Studies reported maternal or paternal preconception exposure, but failed to underscore the exact time and duration. Therefore, there is a need for more rigorous studies to test the hypotheses. With the rising epidemiological trends of smoking, alcohol and illicit drugs consumption in low and middle income countries, it is important that more studies should be conducted in these countries as well.

## Conclusion

Use of caffeine, alcohol, and other illicit drugs and exposure to environmental exposure to radiation and chemical solvents put a significant health risks to the health of women and their children. Identification of substance abuse and environmental history during preconception period provides an opportunity to assist women in reducing major health risks and identify key determinants of healthy pregnancy. Studies have shown that the aversion and prevention of exposure feasibility can play an important role in improving the health of women and their families.

## Competing interests

We do not have any financial or non-financial competing interests for this review.

## Peer review

Peer review reports are included in additional file 1.

## Supplementary Material

Additional file 1 Peer review reports. Click here for file

## Declarations

This article has been published as part of *Reproductive Health* Volume 11 Supplement 2, 2014: Preconception interventions. The full contents of the supplement are available online at http://www.reproductive-health-journal.com/supplements/11/S3. Publication charges for this collection were funded by the Partnership for Maternal, Newborn & Child Health (PMNCH).
