# Supplementary material for: Preconception care: caffeine, smoking, alcohol, drugs and other environmental chemical/radiation exposure
Source: Reprod Health. 2014 Sep 26;11(Suppl 3):S6. doi: 10.1186/1742-4755-11-S3-S6 (PMC4196566; doi:10.1186/1742-4755-11-S3-S6)
Supplement: Additional file 1 — Peer review reports. [file 1742-4755-11-S3-S6-S1.pdf]

Referee's comments to the authors– this sheet WILL be seen by the author(s) and published with the article

Title	**Preconception - substance abuse prevention and life style changes**
Author(s)	Zohra S Lassi, Ayesha M Imam, Sohni V Dean, Zulfiqar A Bhutta
Referee's name	Susan McDonald

When assessing the work, please consider the following points, where applicable:

1. Is the question posed by the authors new and well defined?
2. Are the methods appropriate and well described, and are sufficient details provided to replicate the work?
3. Are the data sound and well controlled?
4. Does the manuscript adhere to the relevant standards for reporting and data deposition?
5. Are the discussion and conclusions well balanced and adequately supported by the data?
6. Do the title and abstract accurately convey what has been found?
7. Is the writing acceptable?

Please make your report as constructive and detailed as possible in your comments so that authors have the opportunity to overcome any serious deficiencies that you find and please also divide your comments into the following categories:

- **Major Compulsory Revisions (which the author must respond to before a decision on publication can be reached)**
- Minor Essential Revisions (such as missing labels on figures, or the wrong use of a term, which the author can be trusted to correct)
- Discretionary Revisions (which are recommendations for improvement but which the author can choose to ignore)

Where possible please supply references to substantiate your comments.

When referring to the manuscript please provide specific page and paragraph citations where appropriate.

Major compulsory revisions:

General comments:

A general observation of the article was that the passion for the topic by the authors was evident. This has perhaps, in my assessment , made the tone of the article sound a little less than objective in parts. It is preferable to stay away from comments such "social evil" and self-inflicted

1. Is the question posed by the authors new and well defined? NO

2. Are the methods appropriate and well described, and are sufficient details provided to replicate the work?

I found the construct of the graphs related to the individual systematic reviews difficult to review as there was not a consistent approach to the information reported in the reviews. The number of participants in each of the studies and or reviews, the quality of the trials, etc is not reported so it is difficult to know how reliable the findings are

(continue on the next sheet)

I feel the authors would benefit from trying to apply a methodology that provides some level of consistency across interpretation of the information

3. Are the data sound and well controlled? NO

As suggested in the previous section, there is a lack of detail about the included studies, particularly the quality and consistency of data collection within each of the areas studied

4. Does the manuscript adhere to the relevant standards for reporting and data deposition?

No, I do not feel the authors perhaps understood all the components necessary to construct a systematic review

5. Are the discussion and conclusions well balanced and adequately supported by the data? Sometimes

The authors discussion and conclusions were based on the outcomes of the tables constructed within the individual topic area reviews, but as it is not certain that the contents of each of these reviews extracted the best or most accurate data, it is possible that the conclusions and discussion are flawed

6. Do the title and abstract accurately convey what has been found? It may be better to include in the title reference to review of the literature related to substance abuse prevention and lifestyle changes

7. Is the writing acceptable? Overall, the writing is very acceptable, however, I think perhaps the authors may have used a thesaurus or dictionary at times which resulted in a few less commonly used English phrases eg *decrement* (page 5) would be more commonly expressed as *decrease, avertable* would be more commonly be expressed as *avoidable, etc,* but these are minor issues

Referee's comments to the authors– this sheet WILL be seen by the author(s) and published with the article

Title	**Preconception - substance abuse prevention and life style changes**
Author(s)	Zohra S Lassi, Ayesha M Imam, Sohni V Dean, Zulfiqar A Bhutta
Referee's name	Susan Sawyer

When assessing the work, please consider the following points, where applicable:

1. Is the question posed by the authors new and well defined?
2. Are the methods appropriate and well described, and are sufficient details provided to replicate the work?
3. Are the data sound and well controlled?
4. Does the manuscript adhere to the relevant standards for reporting and data deposition?
5. Are the discussion and conclusions well balanced and adequately supported by the data?
6. Do the title and abstract accurately convey what has been found?
7. Is the writing acceptable?

Please make your report as constructive and detailed as possible in your comments so that authors have the opportunity to overcome any serious deficiencies that you find and please also divide your comments into the following categories:

- Major Compulsory Revisions (which the author must respond to before a decision on publication can be reached)
- Minor Essential Revisions (such as missing labels on figures, or the wrong use of a term, which the author can be trusted to correct)
- Discretionary Revisions (which are recommendations for improvement but which the author can choose to ignore)

Where possible please supply references to substantiate your comments.

When referring to the manuscript please provide specific page and paragraph citations where appropriate.

General comments:

This paper aimed to undertake a systematic review of (1) pre-conception risks of various exposures, including caffeine, alcohol, tobacco as well as radiation and chemical exposure and (2) effect of interventions to reduce risks.

Major compulsory revisions:

There are a number of major issues I had with this paper :

1. Rational and language around exposures. There was a lack of consistency about the description of the exposure of interest. In the first paragraph alone, the authors refer to 'internal factors' and 'external factors' and then also to 'voluntary' and 'involuntary' exposures. It would be helpful to have a stronger conceptual framework presented to frame why the particular exposures of interest were identified as the focus of the systematic review.
2. Inadequate description of methodology. There is effectively no methods section described. While I gather that this paper is one of a proposed series of publications on preconception care, each paper would still need to stand alone - which this paper doesn't currently do. There are no definitions provided of many of the terms used (eg pre-conception period, peri-conception period). There was similarly little data presented about the severity of the exposure. I was also confused by the language of 'risk aversion' in relation to study type and wondered if the authors mean 'risk reduction'. There was inadequate description of which data bases were searched.

Continued:

3. Inadequate description of the quality of the publications. No description of quality was provided for any of the papers presented. In particular it was very hard to judge from the description of individual papers what was the severity of the exposure and the timing of the exposure in relation to conception.
4. Language. The paper used highly judgemental and overly emotive and generalised language without appropriate referencing (eg in the introduction, 'alcohol use is rampant in women of reproductive age'). Terms were not well defined (eg the term substance abuse used I nthe title but not defined. It is equally used in reference to caffeine as to tobacco and alcohol, but used too loosely.
5. Presentation of results. Within each section, the authors first summarise the health concerns (non systematic review), then review the papers about interventions following which they review and report on the risks of the exposure. This order is most confusing. The introduction within each section should be redundant if a more complete introduction were provided.

Referee's comments to the authors– this sheet WILL be seen by the author(s) and published with the article

Title	**Preconception - substance abuse prevention and life style changes**
Author(s)	Zohra S Lassi, Ayesha M Imam, Sohni V Dean, Zulfiqar A Bhutta
Referee's name	Susan McDonald

When assessing the work, please consider the following points, where applicable:

1. Is the question posed by the authors new and well defined?
2. Are the methods appropriate and well described, and are sufficient details provided to replicate the work?
3. Are the data sound and well controlled?
4. Does the manuscript adhere to the relevant standards for reporting and data deposition?
5. Are the discussion and conclusions well balanced and adequately supported by the data?
6. Do the title and abstract accurately convey what has been found?
7. Is the writing acceptable?

Please make your report as constructive and detailed as possible in your comments so that authors have the opportunity to overcome any serious deficiencies that you find and please also divide your comments into the following categories:

- **Major Compulsory Revisions (which the author must respond to before a decision on publication can be reached)**
- Minor Essential Revisions (such as missing labels on figures, or the wrong use of a term, which the author can be trusted to correct)
- Discretionary Revisions (which are recommendations for improvement but which the author can choose to ignore)

Where possible please supply references to substantiate your comments.

When referring to the manuscript please provide specific page and paragraph citations where appropriate.

Major compulsory revisions:
General comments:

A general observation of the article was that the passion for the topic by the authors was evident. This has perhaps, in my assessment , made the tone of the article sound a little less than objective in parts. It is preferable to stay away from comments such "social evil" and self-inflicted. We have removed these words from the manuscript.

1.Is the question posed by the authors new and well defined? NO We understand that the topic has been studied before, but those were generally limited to content of preconception care for those domain only. In this review we have reviewed the content along with the evidence from the risk aversion studies and from intervention studies as well.

2. Are the methods appropriate and well described, and are sufficient details provided to replicate the work? We have now described the methodology in brief and given the reference to Paper 1 where it has been described fully.

I found the construct of the graphs related to the individual systematic reviews difficult to review as there was not a consistent approach to the information reported in the reviews. The number of participants in each of the studies and or reviews, the quality of the trials, etc is not reported so it is difficult to know how reliable the findings are. We have improved the content and have mentioned in the plots whether the evidence is coming from observational studies or intervention studies. Also the number of partcipants are given in the plots as well. We can also give a hyperlink to the document where all the studies are described and their quality has been assessed.
https://globalmotherchildresearch.tghn.org/site_media/media/articles/Preconception_Report.pdf

I feel the authors would benefit from trying to apply a methodology that provides some level of consistency across interpretation of the information
We have now described the methodology in brief and given the reference to Paper 1 where it has been described fully.

3. Are the data sound and well controlled? NO
As suggested in the previous section, there is a lack of detail about the included studies, particularly the quality and consistency of data collection within each of the areas studied
We have now provided the overall quality assessment in the first paragraph of results section. Also we have provided all the information which we could extract from the paper. Added a limitation section in discussion.

4. Does the manuscript adhere to the relevant standards for reporting and data deposition?
No, I do not feel the authors perhaps understood all the components necessary to construct a systematic review
We have now revisited the paper all over again and redrafted many sections.

5. Are the discussion and conclusions well balanced and adequately supported by the data?
Sometimes
Revisited them and added a new discussion section for the paper.

The authors discussion and conclusions were based on the outcomes of the tables constructed within the individual topic area reviews, but as it is not certain that the contents of each of these reviews extracted the best or most accurate data, it is possible that the conclusions and discussion are flawed
We have now revisited the content. Moved the discussion part from individual section to the end under Discussion section only.

6. Do the title and abstract accurately convey what has been found? It may be better to include in the title reference to review of the literature related to substance abuse prevention and lifestyle changes
Changed the title to be more reflective

7. Is the writing acceptable? Overall, the writing is very acceptable, however, I think perhaps the authors may have used a thesaurus or dictionary at times which resulted in a few less commonly used English phrases eg *decrement* (page 5) would be more commonly expressed as *decrease, avertable* would be more commonly be expressed as *avoidable, etc,* but these are minor issues
We have corrected these now.

Referee's comments to the authors– this sheet WILL be seen by the author(s) and published with the article

Title	**Preconception - substance abuse prevention and life style changes**
Author(s)	Zohra S Lassi, Ayesha M Imam, Sohni V Dean, Zulfiqar A Bhutta
Referee's name	Susan Sawyer

When assessing the work, please consider the following points, where applicable:

1. Is the question posed by the authors new and well defined?
2. Are the methods appropriate and well described, and are sufficient details provided to replicate the work?
3. Are the data sound and well controlled?
4. Does the manuscript adhere to the relevant standards for reporting and data deposition?
5. Are the discussion and conclusions well balanced and adequately supported by the data?
6. Do the title and abstract accurately convey what has been found?
7. Is the writing acceptable?

Please make your report as constructive and detailed as possible in your comments so that authors have the opportunity to overcome any serious deficiencies that you find and please also divide your comments into the following categories:

- Major Compulsory Revisions (which the author must respond to before a decision on publication can be reached)
- Minor Essential Revisions (such as missing labels on figures, or the wrong use of a term, which the author can be trusted to correct)
- Discretionary Revisions (which are recommendations for improvement but which the author can choose to ignore)

Where possible please supply references to substantiate your comments.

When referring to the manuscript please provide specific page and paragraph citations where appropriate.

General comments:

This paper aimed to undertake a systematic review of (1) pre-conception risks of various exposures, including caffeine, alcohol, tobacco as well as radiation and chemical exposure and (2) effect of interventions to reduce risks.

Major compulsory revisions:

There are a number of major issues I had with this paper :

3. Rational and language around exposures. There was a lack of consistency about the description of the exposure of interest. In the first paragraph alone, the authors refer to 'internal factors' and 'external factors' and then also to 'voluntary' and 'involuntary' exposures. It would be helpful to have a stronger conceptual framework presented to frame why the particular exposures of interest were identified as the focus of the systematic review.
   We have now changed these words as suggested by other reviewer as well.
4. Inadequate description of methodology. There is effectively no methods section described. While I gather that this paper is one of a proposed series of publications on preconception care, each paper would still need to stand alone - which this paper doesn't currently do. There are no definitions provided of many of the terms used (eg pre-conception period, peri-conception period). There was similarly little data presented about the severity of the exposure. I was also confused by the language of 'risk aversion' in relation to study type and wondered if the authors mean 'risk reduction'. There was inadequate description of which data bases were searched.
   We have now added methodology. The definitions of preconception and periconception care. Also by risk aversion we meant risk reduction.

Continued:

7. Inadequate description of the quality of the publications. No description of quality was provided for any of the papers presented. In particular it was very hard to judge from the description of individual papers what was the severity of the exposure and the timing of the exposure in relation to conception.
   We have now reported the overall quality of included studies. Severity of the exposure is now been added in the results. The severity of exposure and timing of exposure were not uniformly reported. Hence we have now added this as limitation in the discussion section.
   The quality of the studies can be referred from the document we can provide (for hyperlinking).
8. Language. The paper used highly judgemental and overly emotive and generalised language without appropriate referencing (eg in the introduction, 'alcohol use is rampant in women of reproductive age'). Terms were not well defined (eg the term substance abuse used I nthe title but not defined. It is equally used in reference to caffeine as to tobacco and alcohol, but used too loosely.
   Revisited and removed those judgmental words. Also changes the title.
9. Presentation of results. Within each section, the authors first summarise the health concerns (non systematic review), then review the papers about interventions following which they review and report on the risks of the exposure. This order is most confusing. The introduction within each section should be redundant if a more complete introduction were provided.
   This is how other papers are also drafted. Since we cannot present all the information about that preconception risk in the main introduction therefore we have highlighted them in here along with other inclusion information re that risk.

Referee's comments to the authors– this sheet WILL be seen by the author(s) and published with the article

Title	**Preconception - substance abuse prevention and life style changes**
Author(s)	Zohra S Lassi, Ayesha M Imam, Sohni V Dean, Zulfiqar A Bhutta
Referee's name	Susan McDonald

When assessing the work, please consider the following points, where applicable:

1. **Is the question posed by the authors new and well defined?**

   Acceptable

   2. Are the methods appropriate and well described, and are sufficient details provided to replicate the work?

   I have concerns about the weighting attributed to observational studies that do not assure the reader that the populations studied were homogenous

   3. Are the data sound and well controlled?

   As Above

   4. Does the manuscript adhere to the relevant standards for reporting and data deposition?

   The authors need to be careful in making claims in the abstract and the text that would lead the reader to that definitive links or causation have been found based on the results of this review when the majority of papers are from observational studies and there is little information a=bout the quality of the studies. I raised this point in the original review of the manuscript. I am also a bit confused as to why Figure 4 reported on a non significant result rather than presenting the data for one of only 2 significant results … that of congenital heart defects (OR 2.8 95% CI 1.76-4.47)

   5. Are the discussion and conclusions well balanced and adequately supported by the data?

   The authors have indicated that results for some studies need to be viewed with caution. I would go further and say the results and conclusions in the whole of the paper need to be viewed with extreme caution and highlight the need for more rigorous research to test the hypotheses

   6. Do the title and abstract accurately convey what has been found?

   It may be better to include wording that reflects that this paper is examining the avialble literature for the possible effects caffeine consumption or exposure to chemicals etc may have on the growing fetus

   7. Is the writing acceptable? Yes, the standard of writing is of an acceptable standard

Please make your report as constructive and detailed as possible in your comments so that authors have the opportunity to overcome any serious deficiencies that you find and please also divide your comments into the following categories:

- Major Compulsory Revisions (which the author must respond to before a decision on publication can be reached)
- Minor Essential Revisions (such as missing labels on figures, or the wrong use of a term, which the author can be trusted to correct)
- Discretionary Revisions (which are recommendations for improvement but which the author can choose to ignore)

Where possible please supply references to substantiate your comments.

When referring to the manuscript please provide specific page and paragraph citations where appropriate.

The revised manuscript was a significant improvement. However there are still some issues that require addressing

Moderate compulsory revisions: as outlined above

(continue on the next sheet)

Continued:

This revised paper is greatly improved in some areas. For example, there is now a better description of the methods and the language is less judgmental in tone. There has also been an attempt in the methods to better differentiate the dual focus of the systematic review that sets out to identify both the (1) risk of exposures and (2) the effect of preconceptual interventions. The description of this as a dual focus could still be improved however, and needs to flow better through the rest of the paper.

I continue to have concerns about the presentation and discussion of the results.

1. For example, despite this dual focus for the review, most of the titles for the subsections use language that suggests that the focus is on interventions (eg reducing alcohol exposure, reducing periconceptional caffeine intake, smoking cessation, etc). Yet none of the first 3 figures are on any interventions. Indeed there are NO figures of data about any benefits of interventions. At the very least, the headings of these sections need changing from a primary focus on interventions. For example, the first three subheadings in the result refer to “Reducing periconceptional caffeine intake”, “Reducing alcohol intake” and “Smoking cessation”. While some data is presented about this, all of the figures visualise studies of the effect of the exposure rather than any intervention. In this context, the heading feels misleading.
2. The language remains quite judgmental at times. For example, in the first paragraph of the discussion the text reads, “A growing number of women in their reproductive years continue to consume caffeine, alcohol, tobacco or illicit drugs. While it is distressing that women (and men) continue to put themselves at risk, it is inexcusable that healthcare providers generally do not intervene to ameliorate these risk factors.” Firstly, there has been little emphasis given about the effect of caffeine during pregnancy. Thus, notwithstanding the data presented about caffeine in the paper, for women to be accused of ‘continuing to consume caffeine’ feels quit inappropriate. Similarly, in the absence of intervention studies that health professional interventions might be able to reduce women’s caffeine consumption during pregnancy, it feels highly inappropriate for health care professionals to be lambasted for failing to do their job. This is especially as no data is presented at all about what health professionals do or don’t do in practice – which was anyways not the focus of the review!
3. There continue to be various statements of inappropriate and unsubstantiated claims throughout the paper. For example, in the discussion is the statement, “The number of unplanned pregnancies is on the rise.” This very broad statement (that could refer to a rising rate of pregnancy in all women, in teenage girls, in all parts of the world, in LMIC or in HIC) needs qualifying and referencing. Similarly, in the results under the caffeine section is the very bold statement that, ‘Given the ubiquitous consumption of caffeine in many countries, it is imperative to definitively establish the magnitude of this risk and intervene in preconception period [11-13].’ This statement would feel more appropriate (if toned down) for the discussion, rather than within the very first paragraph of the caffeine section, where purportedly, a neutral position is being taken about the impacts of caffeine in the periconception period. At the very least it feels inappropriate given the lack of data presented about the ubiquity or not of caffeine use across the world.
4. In the discussion, what would be helpful to discuss is the relative strength of evidence. From the data presented, the evidence is weak, not necessarily that there isn’t evidence of much effect, but due to so few studies in this area. What would also be interesting would be a discussion about why the evidence is so weak, and why it might be weaker in some areas than others. And about the potential significance of the associations shown in the context of epidemiological trends for rising rates of, for example, alcohol use and smoking in women being on the rise in LMIC.

Referee's comments to the authors– this sheet WILL be seen by the author(s) and published with the article

Title	**Preconception - substance abuse prevention and life style changes**
Author(s)	Zohra S Lassi, Ayesha M Imam, Sohni V Dean, Zulfiqar A Bhutta
Referee's name	Susan McDonald

When assessing the work, please consider the following points, where applicable:

2. **Is the question posed by the authors new and well defined?**
Acceptable

2. Are the methods appropriate and well described, and are sufficient details provided to replicate the work?
I have concerns about the weighting attributed to observational studies that do not assure the reader that the populations studied were homogenous
We understand the issue around meta-analyzing observational studies. However, not all situation allow randomized controlled trials and sometimes researchers need to estimate results from observational data. For that reason Inverse probability-weighted estimation is a powerful tool for use with observational data. Hence, we used generalized inverse variance to pool data. We have now added this information in the methods section.

3. Are the data sound and well controlled?
As Above
Same as above.

4. Does the manuscript adhere to the relevant standards for reporting and data deposition?
The authors need to be careful in making claims in the abstract and the text that would lead the reader to that definitive links or causation have been found based on the results of this review when the majority of papers are from observational studies and there is little information a=bout the quality of the studies. I raised this point in the original review of the manuscript.
Information related to each included study can be found on the following link:
https://globalmotherchildresearch.tghn.org/site_media/media/articles/Preconception_Report.pdf
We have added this information in the first paragraph of results section.

I am also a bit confused as to why Figure 4 reported on a non significant result rather than presenting the data for one of only 2 significant results … that of congenital heart defects (OR 2.8 95% CI 1.76-4.47).
We have replaced the figure with the one suggested.

5. Are the discussion and conclusions well balanced and adequately supported by the data?
The authors have indicated that results for some studies need to be viewed with caution. I would go further and say the results and conclusions in the whole of the paper need to be viewed with extreme caution and highlight the need for more rigorous research to test the hypotheses
Added the word "great caution" in act and in discussion conclusion section. Also added Therefore, there is a need for more rigorous studies to test the hypotheses." In abstract and discussion section.

6. Do the title and abstract accurately convey what has been found?
It may be better to include wording that reflects that this paper is examining the avialble literature for the possible effects caffeine consumption or exposure to chemicals etc may have on the growing fetus
Added the following in the abstract. "This paper is examining the available literature for the possible effects of caffeine consumption, smoking, alcohol or exposure to chemicals may have on the maternal, newborn and child health (MNCH)."

7. Is the writing acceptable? Yes, the standard of writing is of an acceptable standard
Thanks.

Please make your report as constructive and detailed as possible in your comments so that authors have the opportunity to overcome any serious deficiencies that you find and please also divide your comments into the following categories:

- Major Compulsory Revisions (which the author must respond to before a decision on publication can be reached)
- Minor Essential Revisions (such as missing labels on figures, or the wrong use of a term, which the author can be trusted to correct)
- Discretionary Revisions (which are recommendations for improvement but which the author can choose to ignore)

Where possible please supply references to substantiate your comments.

When referring to the manuscript please provide specific page and paragraph citations where appropriate.

The revised manuscript was a significant improvement. However there are still some issues that require addressing

Moderate compulsory revisions: as outlined above

This revised paper is greatly improved in some areas. For example, there is now a better description of the methods and the language is less judgmental in tone. There has also been an attempt in the methods to better differentiate the dual focus of the systematic review that sets out to identify both the (1) risk of exposures and (2) the effect of preconceptual interventions. The description of this as a dual focus could still be improved however, and needs to flow better through the rest of the paper.

I continue to have concerns about the presentation and discussion of the results.

5. For example, despite this dual focus for the review, most of the titles for the subsections use language that suggests that the focus is on interventions (eg reducing alcohol exposure, reducing periconceptional caffeine intake, smoking cessation, etc). Yet none of the first 3 figures are on any interventions. Indeed there are NO figures of data about any benefits of interventions. At the very least, the headings of these sections need changing from a primary focus on interventions. For example, the first three subheadings in the result refer to "Reducing periconceptional caffeine intake", "Reducing alcohol intake" and "Smoking cessation". While some data is presented about this, all of the figures visualise studies of the effect of the exposure rather than any intervention. In this context, the heading feels misleading.
   We changed all the heading such as "Periconception caffeine intake, pre-and peri-conception smoking, alcohol intake and illicit drugs consumption.
6. The language remains quite judgmental at times. For example, in the first paragraph of the discussion the text reads, "A growing number of women in their reproductive years continue to consume caffeine, alcohol, tobacco or illicit drugs. While it is distressing that women (and men) continue to put themselves at risk, it is inexcusable that healthcare providers generally do not intervene to ameliorate these risk factors." Firstly, there has been little emphasis given about the effect of caffeine during pregnancy. Thus, notwithstanding the data presented about caffeine in the paper, for women to be accused of 'continuing to consume caffeine' feels quit inappropriate. Similarly, in the absence of intervention studies that health professional interventions might be able to reduce women's caffeine consumption during pregnancy, it feels highly inappropriate for health care professionals to be lambasted for failing to do their job. This is especially as no data is presented at all about what health professionals do or don't do in practice – which was anyways not the focus of the review!
   We have now changed the tone and the entire sentence for that matter.
7. There continue to be various statements of inappropriate and unsubstantiated claims throughout the paper. For example, in the discussion is the statement, "The number of unplanned pregnancies is on the rise." This very broad statement (that could refer to a rising rate of pregnancy in all women, in teenage girls, in all parts of the world, in LMIC or in HIC) needs qualifying and referencing. We removed this sentence. Similarly, in the results under the caffeine section is the very

bold statement that, ‘Given the ubiquitous consumption of caffeine in many countries, it is imperative to definitively establish the magnitude of this risk and intervene in preconception period [11-13].’ This statement would feel more appropriate (if toned down) for the discussion, rather than within the very first paragraph of the caffeine section, where purportedly, a neutral position is being taken about the impacts of caffeine in the periconception period. At the very least it feels inappropriate given the lack of data presented about the ubiquity or not of caffeine use across the world. We removed this sentence.

8. In the discussion, what would be helpful to discuss is the relative strength of evidence. From the data presented, the evidence is weak, not necessarily that there isn’t evidence of much effect, but due to so few studies in this area. What would also be interesting would be a discussion about why the evidence is so weak, and why it might be weaker in some areas than others. We observed paucity of studies in each domain; therefore it is difficult to rationalize the reason why the evidence was weaker in one then another. And about the potential significance of the associations shown in the context of epidemiological trends for rising rates of, for example, alcohol use and smoking in women being on the rise in LMIC. Added this point at the end of discussion section.
